# Transcriptome Analysis Reveals the Genetic Basis of the Resveratrol Biosynthesis Pathway in an Endophytic Fungus (*Alternaria* sp. MG1) Isolated from *Vitis vinifera*

**DOI:** 10.3389/fmicb.2016.01257

**Published:** 2016-08-18

**Authors:** Jinxin Che, Junling Shi, Zhenhong Gao, Yan Zhang

**Affiliations:** ^1^College of Food Science and Engineering, Northwest A & F UniversityYangling, China; ^2^Key Laboratory for Space Bioscience and Biotechnology, School of Life Sciences, Northwestern Polytechnical UniversityXi'an, China

**Keywords:** *Alternaria* sp., biosynthesis pathway, genes, resveratrol, transcriptome analysis

## Abstract

*Alternaria* sp. MG1, an endophytic fungus previously isolated from Merlot grape, produces resveratrol from glucose, showing similar metabolic flux to the phenylpropanoid biosynthesis pathway, currently found solely in plants. In order to identify the resveratrol biosynthesis pathway in this strain at the gene level, *de novo* transcriptome sequencing was conducted using Illumina paired-end sequencing. A total of 22,954,434 high-quality reads were assembled into contigs and 18,570 unigenes were identified. Among these unigenes, 14,153 were annotated in the NCBI non-redundant protein database and 5341 were annotated in the Swiss-Prot database. After KEGG mapping, 2701 unigenes were mapped onto 115 pathways. Eighty-four unigenes were annotated in major pathways from glucose to resveratrol, coding 20 enzymes for glycolysis, 10 for phenylalanine biosynthesis, 4 for phenylpropanoid biosynthesis, and 4 for stilbenoid biosynthesis. Chalcone synthase was identified for resveratrol biosynthesis in this strain, due to the absence of stilbene synthase. All the identified enzymes indicated a reasonable biosynthesis pathway from glucose to resveratrol via glycolysis, phenylalanine biosynthesis, phenylpropanoid biosynthesis, and stilbenoid pathways. These results provide essential evidence for the occurrence of resveratrol biosynthesis in *Alternaria* sp. MG1 at the gene level, facilitating further elucidation of the molecular mechanisms involved in this strain's secondary metabolism.

## Introduction

Resveratrol is a phenolic compound that was found to have multiple functions, including neuroprotection, with no major adverse effects (Park and Pezzuto, 2015). High quantities of resveratrol are urgently needed for application in functional food processing (Bhullar and Hubbard, 2015). Currently, resveratrol is mainly produced by extraction from plant materials, which is highly limited by plant growth times and low yields (Kiselev, 2011). Many efforts have been made to construct resveratrol-producing *Escherichia coli* or yeast strains through genetic modification (Kiselev, 2011). Nearly all genes used in currently reported methods are plant based. Identifying microbial genes would provide new genes for metabolic engineering of resveratrol production using microorganisms and thus have great potential in resveratrol production. However, the microbial resveratrol biosynthesis pathway has not yet been identified in the literature.

**Figure 1:**
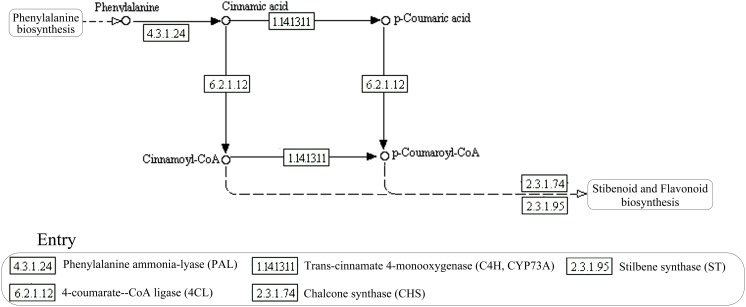
**Schematic representation of the resveratrol biosynthesis pathway in plants**.

Currently, the resveratrol biosynthesis pathway is only found in plants (Figure 1; Tan et al., 2012; Tavares et al., 2013). The key enzymes of this pathway, in order, are phenylalanine ammonia-lyase (PAL), cinnamic acid 4-hydroxylase (CYP73A), 4-coumarate-CoA ligase (4CL), and stilbene synthase (STS), resveratrol synthase (ST), or chalcone synthase (CHS). Phenylalanine ammonia, cinnamic acid, and 4-coumarate-CoA are the key intermediates, in order (Sparvoli et al., 1994; Tan et al., 2012; Tavares et al., 2013). Deamination of phenylalanine, catalyzed by PAL, is the first step in the pathway, followed by the conversion of trans-cinnamate into 4-coumaroyl-CoA, catalyzed by CYP73A and 4CL. Biosynthesis of resveratrol from one 4-coumaroyl-CoA and three malonyl-CoA units is the last step in the pathway and is catalyzed by CHS/ST (Jeandet et al., 2002). The key enzymes of the resveratrol biosynthesis pathway have been found in some microorganisms, such as PAL in some yeast strains (Xue et al., 2007; Liu et al., 2015), CHS/ST in *Saccharopolyspora erythraea* (Magdevska et al., 2010), and 4CL in *Aspergillus clavatus* (Accession No. XM001274066, journal unpublished). Although, these key enzymes were identified in microorganisms separately, the complete biosynthesis pathway has not been predicted in any strain. We previously found *Alternaria* sp. MG1, an endophytic fungus isolated from Merlot grape, could produce resveratrol *in vitro* (Shi et al., 2012). Metabolic flux and some enzymatic activities related to resveratrol biosynthesis were also detected in this strain (Zhang et al., 2013b). However, the enzymatic activity of key enzymes in the pathway, such as STS, ST, or CHS has not been identified before now due to the shortage of proper measurement methods. Therefore, identification of the candidate genes in this strain would be helpful in understanding the resveratrol biosynthesis pathway.

Currently, transcriptome sequencing is an effective approach for mining genetic information from unknown genome sequences, and is useful in gene sequencing, new gene discovery, and studying gene regulation (Wang et al., 2009). This method can produce millions of short cDNA reads and provide useful information on gene expression, based on RNA abundance (Peng et al., 2014; Zhang et al., 2015). Next-generation sequencing technologies such as Roche 454 and Illumina RNA-Seq have emerged during a golden age of scientific discovery (Margulies et al., 2005; Metzker, 2010). The Roche 454 platform is capable of generating 80–120 Mb of sequence data in 200–300-bp reads during a 4 h run, and has seen wide usage in recent times. For microorganisms, the transcriptome analysis of *Pythium ultimum* (Cheung et al., 2008), *Sclerotium rolfsii* (Schmid et al., 2010), and *Golovinomyces* (Wessling et al., 2012) have been successfully performed using the Roche 454 platform. However, the pyrosequencing approach is prone to errors that result from incorrectly estimating the length of homopolymeric sequence stretches. As of 2015, 454 pyrosequencing is nearing retirement and will not be supported by the distributing company (Roche) after 2016 (Bleidorn, 2016). In recent years, a newly developed platform (Illumina Hiseq 2000/2500) has been successfully used in *de novo* sequencing experiments, providing a new transcriptome sequencing approach for non-model species, especially fungal species, without a reference genome (Zhang et al., 2013a; Liu et al., 2014; Lou et al., 2014). This platform can produce a large amount of genetic information for many analysis types, such as comparative transcriptome analysis (Quan et al., 2015; Qiu et al., 2016), SSR identification (Yu et al., 2014; Huang et al., 2015) and uncovering unknown biosynthesis/metabolism pathways, in particular (Zhang et al., 2015; Zondag et al., 2016). Compared with the 454 technology and other sequencing platforms, the Illumina platform has optimized the traditional sequencing method, and is advantageous in its high throughput, high sensitivity, high accuracy and low cost, distinguishing it from its competitors. Based on the price, accuracy, and RNA-seq throughput, using the Illumina platform for this research is a sensible choice.

Therefore, in order to obtain basic information on potential genes in the resveratrol biosynthesis pathway of *Alternaria* sp. MG1, total mRNA was extracted from the strain at high resveratrol-producing stages and then used for RNA-seq library construction. The obtained sequencing data were subsequently subjected to the following analyses: *de novo* assembly of unigenes, bioinformatics analysis, functional annotation, GO classification, and KEGG pathway analysis. The obtained results revealed molecular information regarding gene sequence, identity, and function. Most importantly, the resveratrol biosynthesis pathway was constructed and illustrated from these identified genes.

## Materials and methods

### Materials and RNA extraction

*Alternaria* sp. MG1 (code: CCTCC M 2011348), a strain previously isolated from the cob of Merlot grape, was used in the study and stored in the China Center for Type Culture Collection (Wuhan, China; Shi et al., 2012). For preparation of *Alternaria* sp. MG1 cells, the strain was cultivated in a 250 mL flask containing 100 mL liquid potato-dextrose broth [PDB, 200 g potato (peeled and diced), 20 g glucose, 1000 mL water]. The cultivation was carried out at 28⋅C and 120 rpm in a rotary shaker. After a cultivation time of 21 h, the optimum period previously found to produce abundant resveratrol (Zhang et al., 2013b), the cells were collected by centrifugation at 1136 × *g* for 10 min at 4⋅C using a refrigerated centrifuge (HC-3018R, Anhui USTC Zonkia Scientific Instruments Co., Ltd., Anhui, China). After washing twice with sterile water, the collected cells were immediately stored in liquid nitrogen until further analysis. Extraction of the total RNA from the cells was performed using a Spin Column Fungal total RNA Purification Kit [Sangon Biotech (Shanghai) Co., Ltd., China]. RNA extraction quality and quantity was analyzed using a NanoDrop 2000 Spectrophotometer (Thermo Scientific, Massachusetts, USA) gel electrophoresis, and an Agilent 2100 Bioanalyzer (Agilent Technologies, California, USA).

### mRNA-seq library construction for illumina sequencing

After total RNA extraction, the RNA-seq library was constructed according to the previously reported method (Tang et al., 2014). A reverse transcription-PCR system (Promega, Madison, WI, USA) was used to generate the first DNA strand, and an amplification reaction containing RNase H, DNA polymerase I, and dNTPs was used to produce the second and subsequent DNA strands. A QIAquick purification kit was used to ligate the adaptor sequences to the ends of their double-stranded cDNA. In this way, the library was prepared from 200 to 250 bp (average size, 230 bp) size selected cDNA fragments. The library was subsequently sequenced using the Illumina HiSeq 2500 Platform (Illumina Inc., San Diego, CA, USA) to generate 101 bp paired-end reads.

### Sequence data analysis and assembly

Based on sequencing by synthesis (SBS) technology, the quality reads were assembled using the Illumina HiSeq 2500 platform. The quality score of the raw reads was set at Q30 or above. Generally, the raw reads contain tiny minority primer sequences, adapter sequences, and other potential contaminants. Prior to subsequent analysis, the clean reads were filtered from the raw reads by removing the reads with only adaptor and unknown nucleotides. Data analysis and base calling were performed using Illumina instrument software. Raw sequence data were subsequently deposited in the NCBI database with the SRA study accession number, SRP060338. The trimmed reads obtained for the samples were then assembled into unigenes using the Trinity platform^1^ with the K-mer = 25 and group pairs distance = 500 (maximum length expected between fragment pairs; Grabherr et al., 2011).

### Gene expression analysis and unigenes annotation

To evaluate the expression level of all reads, each unigene was compared with the unigene database by using Bowtie (http://bowtie-bio.sourceforge.net/bowtie2/manual.shtml) and normalized into RPKM (Reads per kb per million reads) values according to formula (1).

(1) $$\text{RPKM}=\frac{\text{Map theunigenereads}}{\begin{array}{l} \text{Mapallunigenesreads}\left(\text{million}\right)\;\times \text{ Theunigenelength}\left(\text{kb}\right) \end{array}}$$

Functional annotation of each unigene was searched against various protein databases, and identified by referring to the annotation information of the given unigene that has the highest sequence similarity with the tested one. All unigenes were also searched against the NCBI non-redundant protein database (Nr), Swiss-Prot, TrEMBL, and Cluster of Orthologous Groups of proteins (COG) using BLAXTX, and against the NCBI nucleotide database (Nt) using BLASTN, with a cut-off E-value of 10^−5^ for both. The Blast2 GO program and WEGO software were also used to obtain the gene ontology (GO) annotations and the distribution of gene functions for each unigene using a cut-off value of 10^−5^ (Conesa et al., 2005; Ye et al., 2006). Assignment of each unigene to different pathways was performed by searching against the Kyoto encyclopedia of genes and genomes (KEGG databases) using BLASTX and KEGG automatic annotation server (KAAS^2^; Moriya et al., 2007).

### Phylogenetic analysis

To align the sequences and construct the phylogenetic tree, the chalcone synthase amino acid sequences of *Alternaria* sp. MG1 were translated from a nucleotide sequence using a web translate tool^3^. A phylogenetic tree was constructed with MEGA (Molecular Evolutionary Genetics Analysis) software version 5.0^4^ using the minimum-evolution method with bootstrapping (1000 replicates). Amino sequence alignment was performed using ClustalW (opening = 10, extension = 0.2), and displayed using GENEDOC version 2.6.002^5^.

## Results

### Sequence analysis and assembly

To obtain an appropriate overview of the transcriptome and gene activity at nucleotide resolution of *Alternaria* sp. MG1, a mixed cDNA sample representing the high resveratrol-producing phase of this strain was prepared and sequenced. Each sequenced sample yielded 2 × 100 bp independent reads from either end of a cDNA fragment. After stringent quality assessment and data filtering, 22,954,434 reads (4.64 Gb) with 53.73% (GC%) and 89.42% Q30 bases (those with a base quality higher than 30) were selected as high quality reads for further analysis (Table 1).

**Table 1:** **Illumina transcriptome sequencing summary for *Alternaria* sp. MG1**.

**Sample**	**Read length**	**Read number**	**Base number**	**GC (%)**	**%≥Q30 (%)**
MG1	PE100	22,954,434	4,636,177,982	53.73	89.42

By using the Trinity *de novo* assembly program, the distribution of data assembly was obtained and shown in Table 2. It was found that all high-quality reads produced 2,010,245 contigs with an N50 length of 45 bp and a mean length of 45.84 bp. A total of 37,163 transcripts were obtained with an N50 length of 5004 bp and a mean length of 2358.86 bp. In total, there were 21,020 transcripts longer than 1 kb and 14,042 transcripts longer than 2 kb. Finally, 18,570 unigenes were identified from the contigs data set with an N50 length of 2153 bp and a mean length of 1122.38 bp, of which 6471 genes (34.85%) were >1 kb. These results indicated that the obtained transcriptome data were acceptable and reliable in quality.

**Table 2:** **Assembled data for contigs, transcripts, and unigenes in the transcriptome of *Alternaria* sp. MG1**.

**Length range**	**Contigs**	**Transcripts**	**Unigenes**
200–300	1,996,791 (99.33%)	5850 (15.74%)	5170 (27.84%)
300–500	3842 (0.19%)	4894 (13.17%)	3755 (20.22%)
500–1000	3062 (0.15%)	5399 (14.53%)	3174 (17.09%)
1000–2000	3201 (0.16%)	6978 (18.78%)	3298 (17.76%)
2000+	3349 (0.17%)	14,042 (37.78%)	3173 (17.09%)
Total number	2,010,245	37,163	18,570
Total length	92,148,728	87,662,222	20,842, 526
N50 length	45	5004	2153
Mean length	45.84	2358.86	1122.38

### Unigenes expression analysis and functional annotation

**Figure 2:**
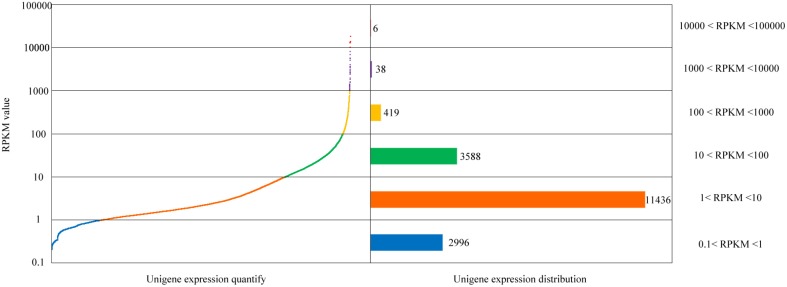
**Quantification and distribution of gene expression in the *Alternaria* sp. MG1 unigenes**.

After comparison of sequencing reads and the unigene database, identification, and functional annotation of unigene expression was obtained. By uniting RNA-Seq by Expectation-Maximization (RSEM) with the comparison results, the gene expression level was estimated. Here, the RPKM values were used to represent the expression abundance of each unigene. The distribution of each gene's expression is shown in Figure 2.

Several complementary approaches were also used to annotate the assembled sequences, including aligning the sequences with those deposited in diverse databases such as Nr, Nt, Swiss-Prot, COG, GO, and KEGG. The best alignment was selected from matches with an E-value of < 10^−5^. The obtained overall functional annotation is depicted in Table 3. In total, 14,186 unigenes were successfully annotated in the transcriptome database, including 11,201 unigenes with a length >300 nt and 6291 unigenes with a length >1000 nt. Due to a unigene may be annotated in more than one database, the total annotated number was less than the sum of all database. The functional annotation of each database is detailed below.

**Table 3:** **Functional annotation of the *Alternaria* sp. MG1 transcriptome**.

**Annotated database**	**Annotated number**	**300–1000 bp**	**≥1000 bp**
COG_annotation	4954	1386	2943
GO_annotation	8190	2595	3958
KEGG_annotation	2701	683	1696
KOG_annotation	6306	1713	3881
Pfam_annotation	7823	2140	4851
Swissprot_annotation	5341	2216	3543
TrEMBL annotation	8201	2216	5051
Nr_annotation	14,153	4983	6297
Total	14,186	4994	6299

**Figure 3:**
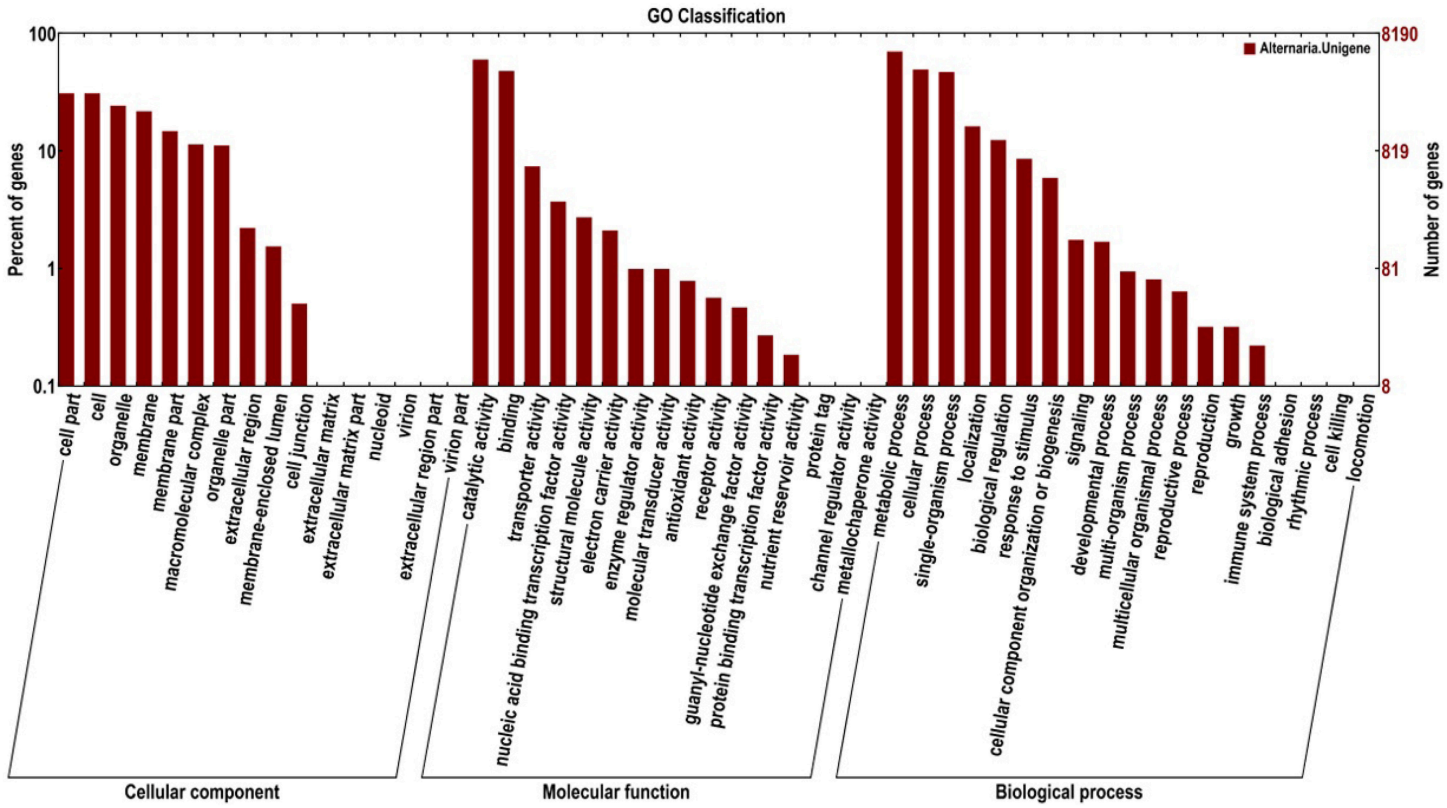
**Functional annotation of the assembled sequences based on gene ontology (GO) categorization**.

The Gene Ontology (GO) project is a collaborative effort to address the need for consistent descriptions of gene products across databases. A further functional classification of all unigenes was performed using a set of plant-specific GO slims (Figure 3). A total of 8190 unigenes (5773%) were assigned to 51 functional groups using GO assignments, including cellular component, molecular function, and biological process. For the three major GO categories, cellular component (12,265 unigenes), molecular function (10,491 unigenes), and biological process (17,720 unigenes), the dominant subcategories were metabolic process (5749 unigenes), catalytic activity (4911 unigenes), cellular process (4047 unigenes), and binding (3926 unigenes), respectively. Cell part (2534 unigenes), organelle (1989 unigenes), biological regulation (1013 unigenes), and transporter activity (605 unigenes) were also well represented. However, a few genes were assigned to the categories cell killing (1 unigene), channel regulator activity (1 unigene), and metallochaperone activity (1 unigene).

**Figure 4:**
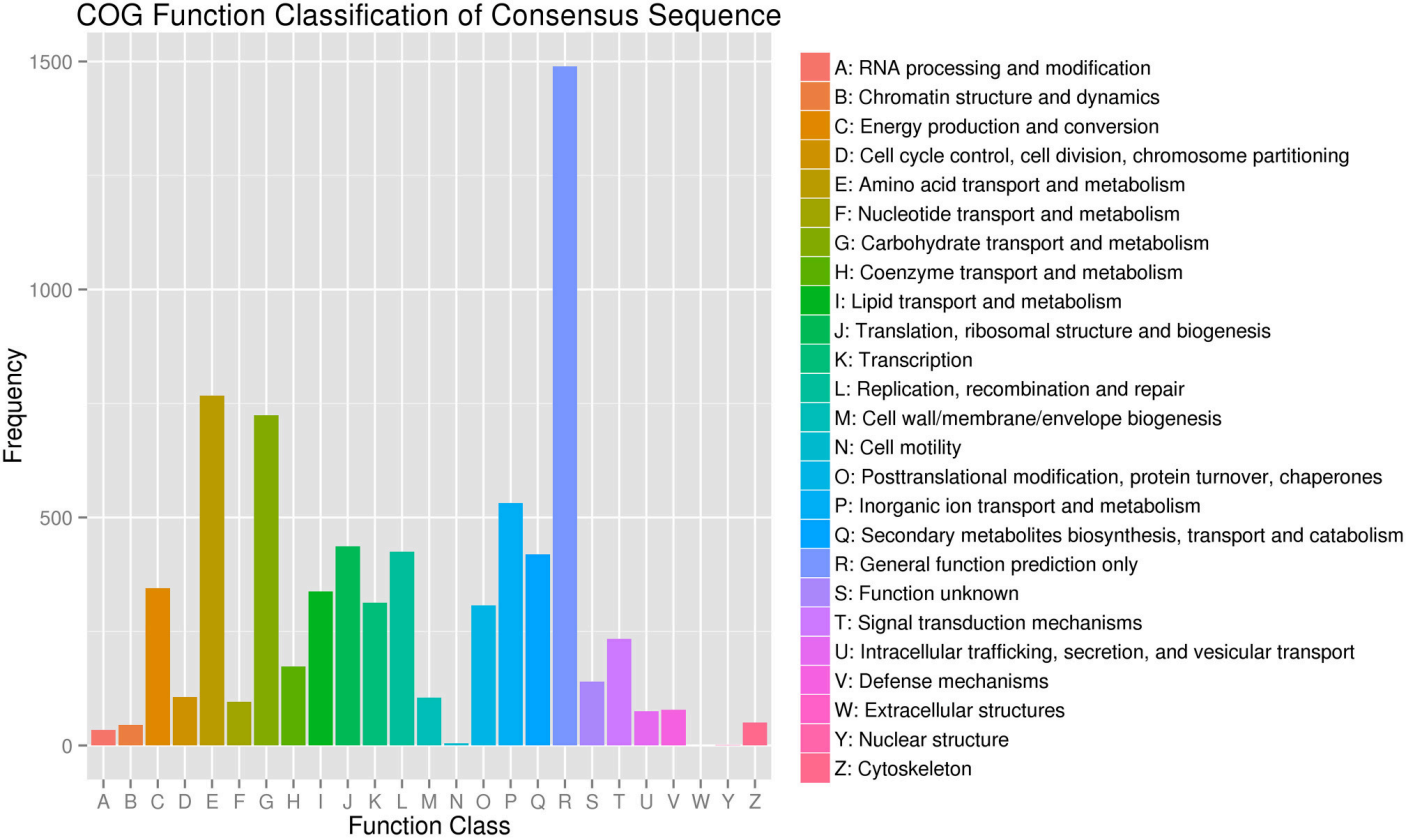
**Classification of the clusters of orthologous groups (COG)**.

To evaluate the completeness of our transcriptome library and the effectiveness of our annotation process further, we used the annotated unigene sequences to search for the genes in the COG classifications. All unigenes were subjected to a search against the COG database for functional prediction and classification. Overall, 4954 sequences showing a hit with the Nr database were successfully assigned to COG classifications (Figure 4). COG annotated putative proteins were functionally classified into at least 25 protein families. The cluster for general function prediction (1489; 30.06%) represented the largest group, followed by amino acid transport and metabolism (767; 15.48%), carbohydrate transport and metabolism (724; 14.61%), inorganic ion transport and metabolism (532; 10.74%), translation, ribosomal structure, and biogenesis (437; 8.82%), replication, recombination, and repair (425; 8.58%), energy production and conversion (345; 6.96%), lipid transport and metabolism (338; 6.82%), transcription (313; 6.32%), post translational modification, protein turnover, and chaperones (307; 6.20%). Only a few unigenes were assigned to cell motility and nuclear structure (5 and 1 unigenes, respectively). No unigene was found in extracellular structure. In addition, 419 (8.46%) unigenes were assigned to secondary metabolite biosynthesis, transport and catabolism, and coenzyme transport and metabolism (173; 3.49%).

To demonstrate the potential application of the generated unigenes of *Alternaria* sp. MG1 in the present study, biochemical pathways were also represented by the unigene collection. Annotations of the obtained unigenes were fed into the KEGG Pathway Tools, an alternative approach to gene function categorization, with an emphasis on biochemical pathways. This process predicted 115 pathways represented by 2701 unigenes (Table 4). Most pathways fell into the categories “metabolic pathways,” “genetic information processing,” “environmental information processing,” “cellular processes,” and “organismal systems.”

**Table 4:** **Pathway classification of *Alternaria sp.* MG1 unigenes**.

**Category**	**Pathway**	**Count**
Metabolism	Amino acid metabolism	379
	Biosynthesis of other secondary metabolites	58
	Carbohydrate metabolism	491
	Energy metabolism	145
	Glycan biosynthesis and metabolism	84
	Lipid metabolism	242
	Metabolism of cofactors and vitamins	106
	Metabolism of other amino acids	100
	Metabolism of terpenoids and polyketides	35
	Nucleotide metabolism	164
Genetic information processing	Translation	420
	Folding, sorting and degradation	39
	Transcription	52
	Replication and repair	46
	Transcription	111
	Folding, sorting, and degradation	47
	Replication and repair	137
	Folding, sorting, and degradation	182
Environmental information processing	Membrane transport	18
	Signal transduction	22
Cellular processes	Transport and catabolism	168
Organismal systems	Immune system	8
	Environmental adaptation	20

The annotated glycan biosynthesis and metabolism pathways (84 unigenes) were “N-glycan biosynthesis” (41 unigenes) and “various types of N-glycan biosynthesis” (11 unigenes), “glycosaminoglycan degradation” (2 unigenes), “glycosylphosphatidylinositol (GPI)-anchor biosynthesis” (21 unigenes), and “glycosphingolipid biosynthesis” (11 unigenes), and “other glycan degradation” (9 unigenes). The annotated pathways involved in terpenoid and polyketide metabolism (35 unigenes) were “terpenoid backbone biosynthesis” (14 unigenes), “limonene and pinene degradation” (11 unigenes), “diterpenoid biosynthesis” (2 unigenes), and “carotenoid biosynthesis” (8 unigenes). Metabolism pathways for the biosynthesis of secondary metabolites (58 unigenes) were also annotated as “phenylpropanoid biosynthesis” (23 unigenes), “flavonoid biosynthesis” (7 unigenes), “flavone and flavomol biosynthesis” (3 unigenes), “stilbenoid, diarylheptanoid, and gingerol biosynthesis” (3 unigenes), “isoquinoline alkaloid biosynthesis” (10 unigenes), “caffeine metabolism” (4 unigenes), and “glucosinolate biosynthesis” (1 unigene).

### Key genes annotated in resveratrol biosynthesis pathway

**Figure 5:**
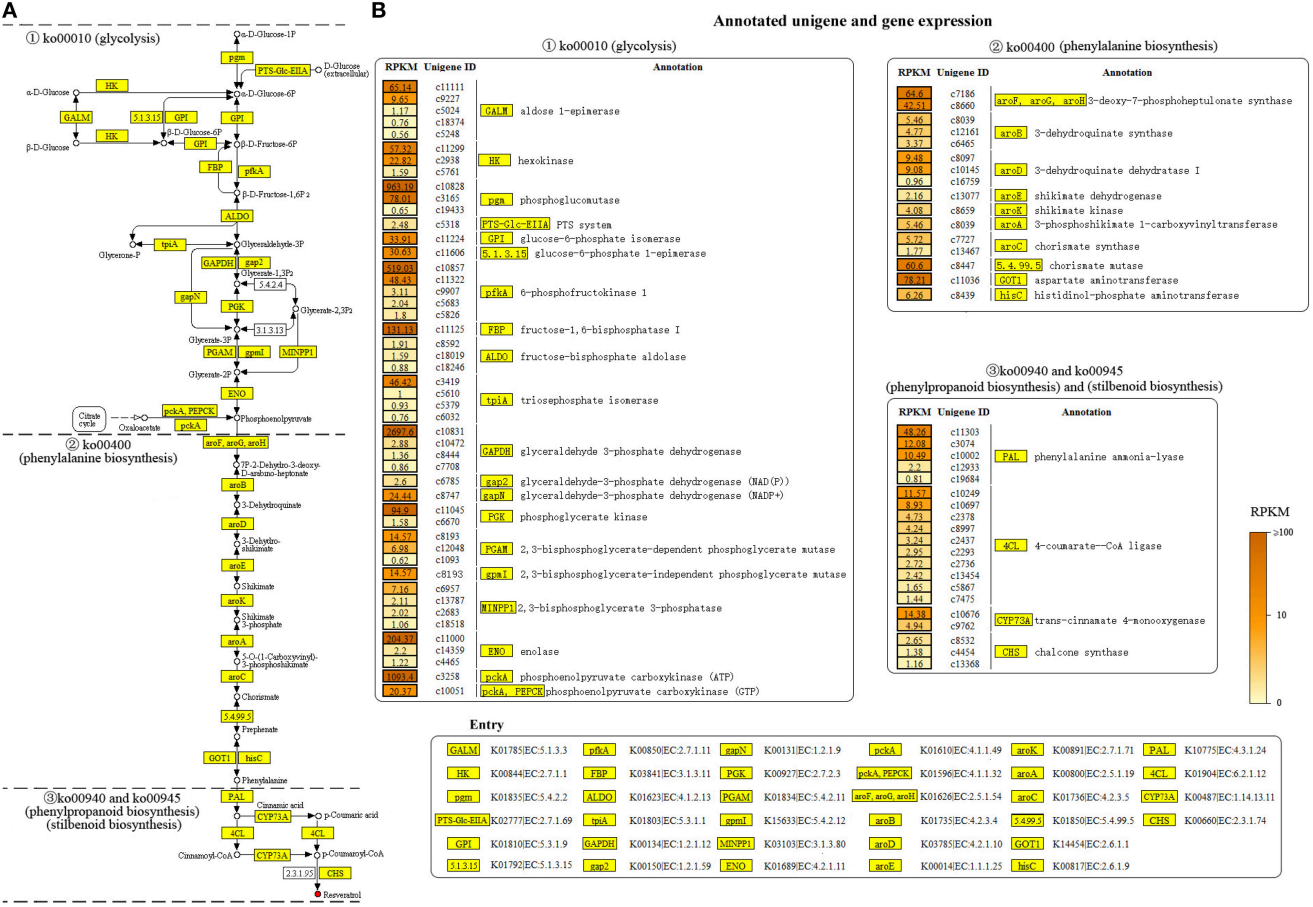
**Annotated unigenes (A) and the level of gene expression (B) involved in resveratrol biosynthesis pathway annotated in *Alternaria* sp. MG1**.

In order to reveal the genetic basis of the resveratrol biosynthesis pathway in *Alternaria* sp. MG1, all unigenes involved in resveratrol biosynthesis were specifically selected in the study. As expected, many genes involved in the pathway were successfully annotated in COG and KEGG databases. A total of 84 unigenes were annotated in the four major metabolism pathways leading to the formation of resveratrol, including glycolysis (ko00010), phenylalanine biosynthesis (ko00400), phenylpropanoid biosynthesis (ko00940), and stilbenoid biosynthesis (ko00945). The annotated unigenes and corresponding RPKM values are indicated in Figure 5.

Glycolysis (ko00010) contributes to the early stage of resveratrol biosynthesis by converting different substrates (e.g., α-D-glucose and β-D-glucose) to phosphoenolpyruvic acid (PEP; Figure 5A-1). In total, 48 unigenes, coding 20 enzymes such as HK, PGI, pfkA, ALDO, tpiA, GADPH, PGK, PGAM, and ENO were identified in this pathway. The unigene expression analysis showed the annotated unigenes encoding these enzymes exhibited a wide range of abundance variation (Figure 5B-1). For example, c10831 and c3258 showed an abundance of 2697.6 and 1093.4 RPKM in GADPH and pfkA, respectively. However, c5248 and c1093 had abundances of only 0.56 and 0.62 RPKM, respectively. Such great deviations in the gene expression level were also observed in different unigenes encoding the same enzyme. For example, in the two unigenes encoding the enzyme HK, c11299 had an abundance of 57.32 RPKM, whereas c5761 had an abundance of 1.59 RPKM only.

Meanwhile, 16 unigenes were annotated in phenylalanine biosynthesis (ko00400; Figure 5A-2). This pathway is essential for the biosynthesis of resveratrol by shikimic acid formation and subsequent conversion to l-phenylalanine. A single unigene encoding one enzyme was widely found in this pathway. The annotated 16 unigenes encoded 10 key enzymes in the pathway, with the unigene expression levels ranging from 0.96 to 78.21 RPKM. The abundance of the key unigenes were 78.21 for GOT1, 64.6 for aroF, 60.6 for chorismate synthase, 9.48 for aroD, 6.26 for hisC, 5.46 for aroB, and RPKM < 5 for the other enzymes.

Finally, key genes contributing to phenylpropanoid biosynthesis and stilbenoid biosynthesis, the most important pathways for resveratrol biosynthesis were also identified in the study. Overall, 20 unigenes (Supplementary Material) encoding four enzymes were annotated in the two pathways for converting L-phenylalanine into resveratrol (Figure 5A-3). Important enzymes in phenylpropanoid biosynthesis (ko00940) and stilbenoid biosynthesis (ko00945) were successfully annotated in our transcriptome database. The expression of the unigenes encoding essential enzymes for resveratrol biosynthesis, PAL, 4CL, and CYP73A were 48.6, 11.57, and 14.38 RPKM, respectively (Figure 5B-3). Resveratrol synthase, the key enzyme catalyzing the last step of resveratrol biosynthesis in plants, was not annotated in the study. However, chalcone synthase (CHS) was successfully annotated in our transcriptome database. CHS was reported as an isoenzyme of resveratrol synthase, having activity on resveratrol biosynthesis, but with low efficiency (Lanz et al., 1991; Jeandet et al., 2010). Three unigenes were annotated for encoding CHS in this study, and their expression levels were 2.65 (c8532), 1.38 (c4454), and 1.16 (c13368) RPKM.

### Phylogenetic analysis of annotated chalcone synthase

**Figure 6:**
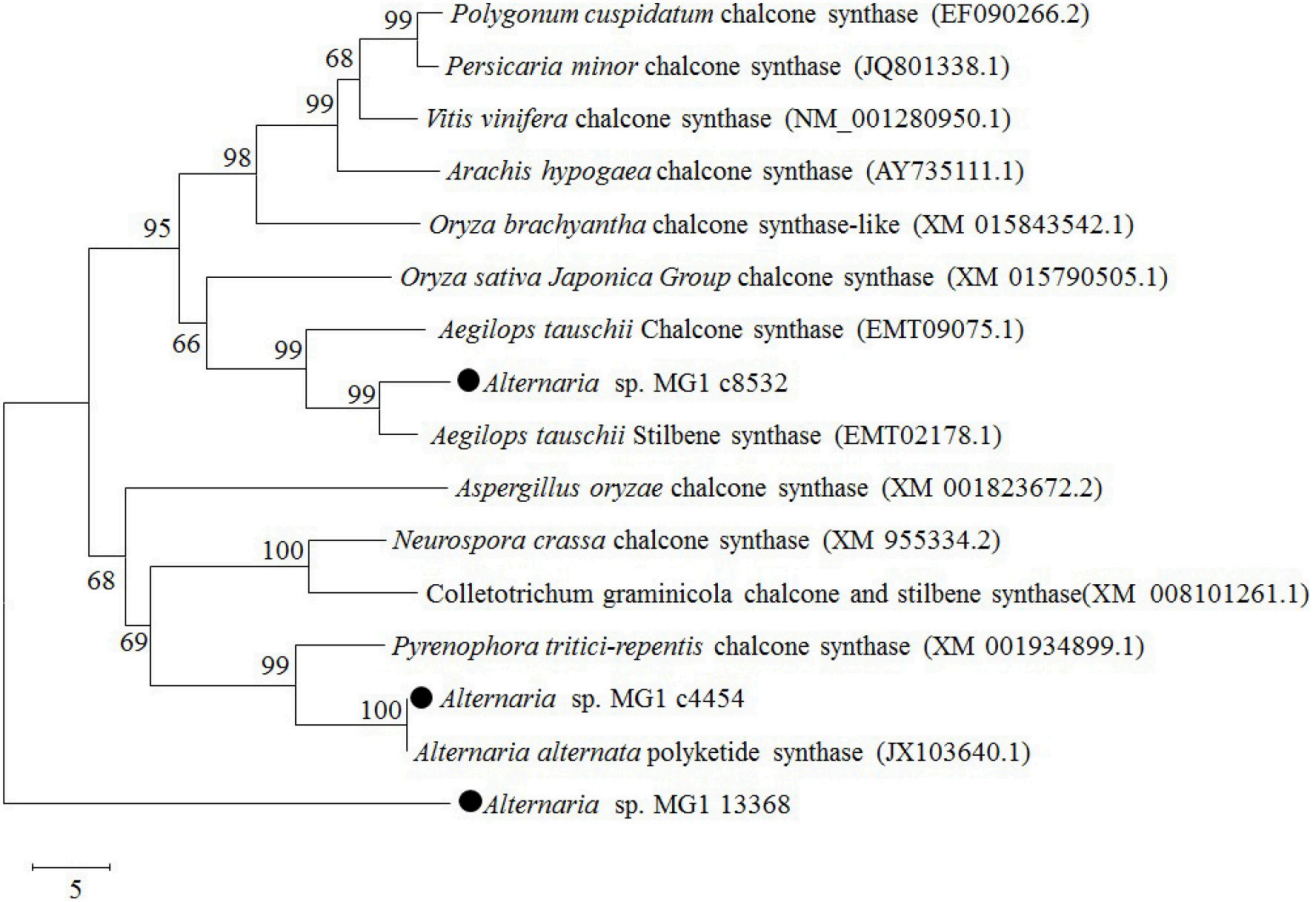
**Phylogenetic relationships of chalcone synthase between *Alternaria* sp. MG1 and other known species using a minimum evolution phylogeny test and 1000 bootstrap replicates**.

Chalcone synthase was reported as an isoenzyme of resveratrol synthase having low activity in resveratrol biosynthesis (Lanz et al., 1991; Jeandet et al., 2010). Three chalcone synthases were annotated in the obtained transcriptome database. They were c8532 (GEMY01016815), c4454 (GEMY01012697), and c13368 (GEMY01001602), with fragment lengths of 1153, 348, and 447 bp and predicted protein sequences of 314, 115, and 149 amino acids, respectively. According to the BLASTP results, c8532 and c4454 had high sequence homology with chalcone synthase and stilbene synthase from different species. Phylogenetic analysis of the CHS-like domain revealed that c8532 and c4454 were located in the same cluster of chalcone and stilbene synthase, while c13368 was located in another outgroup (Figure 6). Comparatively, c8532 was closer to *Aegilops tauschii* stilbene synthase (EMT02178.1) and *A. tauschii* chalcone synthase (EMT09075.1) than other enzymes, while c4454 was similar to *Alternaria alternata* polyketide synthase (JX103640) and *Pyrenophora tritici-repentis* chalcone synthase (XM001934899).

**Figure 7:**
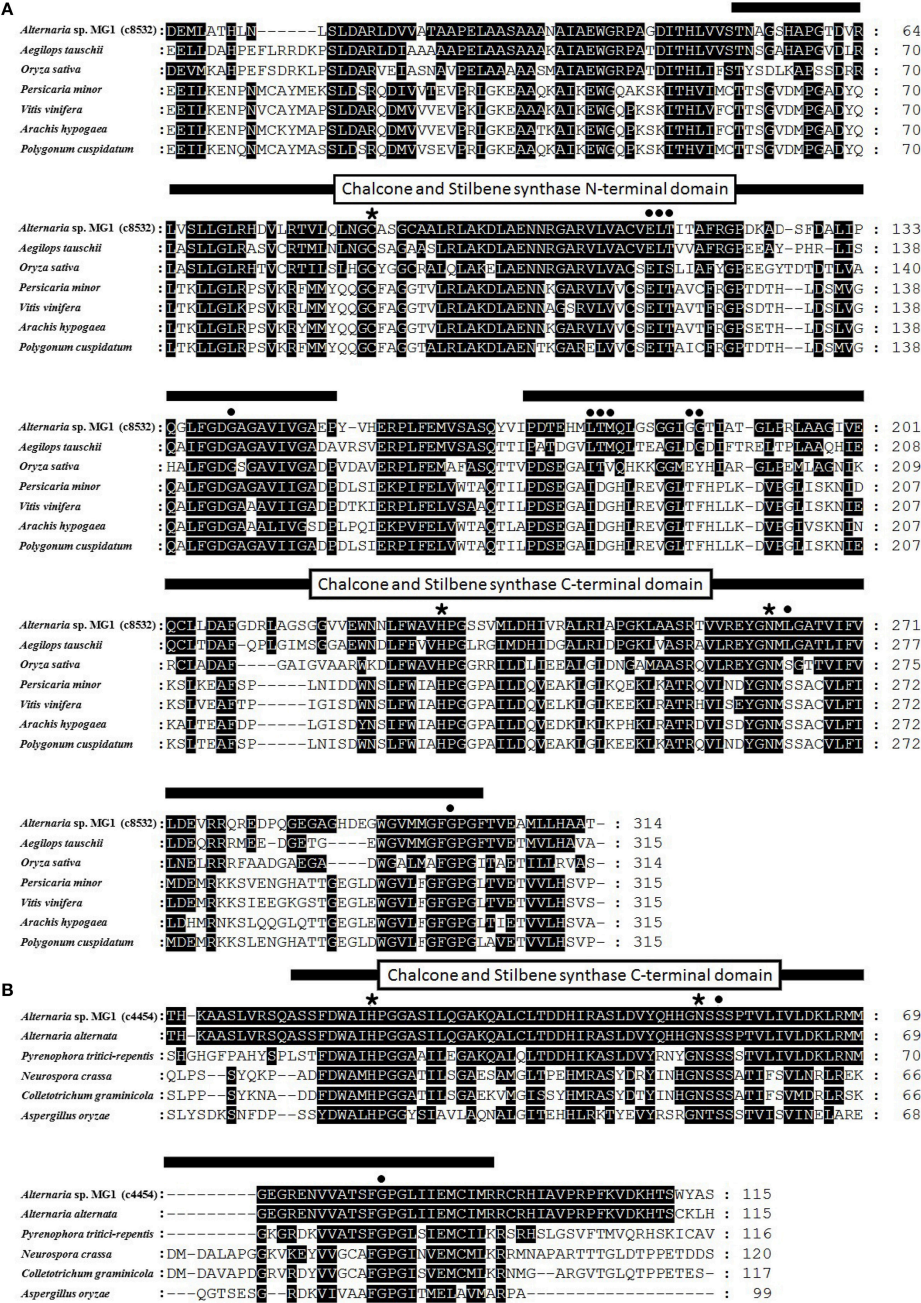
**Comparison of deduced *Alternaria* sp. MG1 CHS proteins with corresponding sequences of other CHS proteins**. Amino acids identical to *Alternaria* sp. MG1 are highlighted in black. Numbers indicate the amino acid positions. Black asterisks indicate active sites, and black dots indicate product binding sites. **(A)** *Alternaria* sp. MG1 c8532 comparison with CHS amino acids sequences from *Aegilops tauschii* (EMT09075.1), *Oryza sativa* (XM015790505.1), *Persicaria minor* (JQ801338.1), *Vitis vinifera* (NM001280950.1), *Arachis hypogaea* (AY735111.1), and *Polygonum cuspidatum* (EF090266.2). **(B)** *Alternaria* sp. MG1 c4454 comparison with *Alternaria alternata* (JX103640.1), *Pyrenophora tritici-repentis* (XM001934899.1), *Neurospora crassa* (XM955334), *Colletotrichum graminicola* (XM008101261.1), and *Aspergillus oryzae* (XM001823672.2).

As shown in Figure 7A, c8532 CHS-like domains were aligned with the corresponding sequences of many reported CHS proteins. There was a more than 50% similarity between c8532 and *A. tauschii* chalcone synthase (68%) and *Oryza sativa* chalcone synthase (54%). The sequence homology of c8532 with selected proteins was 42% for *Persicaria minor*, 45% for *Vitis vinifera*, 43% for *Arachis hypogaea*, and 42% for *Polygonum cuspidatum*. c4544 had the highest homology with *Alternaria alternate* polyketide synthase (96%), and less homology with *P. tritici-repentis* (78%), *Neurospora crassa* (49%), *Colletotrichum graminicola* (47%), and *Aspergillus oryzae* (45%) (Figure 7B). The two CHS-like domains, N-terminal domain and C-terminal domain of chalcone synthase and stilbene synthase were marked in the sequence alignment (Figure 7). The amino acids indicated by a black asterisk were the conserved domains for the active site. The product binding sites were marked with a black dot. Overall, the phylogenetic analysis indicated that c8532 and c4454 might have the same function as chalcone and stilbene synthase.

## Discussion

As a newly explored biological resource, endophytic fungi offer an excellent opportunity to produce active compounds without the same limitations as plant resources. However, critical obstacles such as low and unstable production of metabolites greatly inhibit the application of these fungi (Zhang et al., 2015). Understanding the biosynthesis pathway and relative key genes is essential for an intensive control of their viability and stability. To our knowledge, many resveratrol-producing endophytic fungi have been found in some plants (Donnez et al., 2009; Mei et al., 2015). However, the genomics, transcriptome data, or pathway for resveratrol biosynthesis in microorganisms (including endophytic fungi) has not been elucidated before now.

*Alternaria* is a genus of ascomycete fungi. Many strains of this genus have been identified as saprophytic and as major plant pathogens (Dang et al., 2015). *Alternaria* species have been found to be a widespread, naturally occurring fungal flora. Approximately 95% of species within this genus were found to be facultative parasites in plants (Neergaard, 1945; Pedras et al., 2009). Some species of *Alternaria* were found not only to be responsible for huge economic losses in the agricultural industry (Andersen et al., 2015; Ntasiou et al., 2015), but also directly responsible for the development of severe and potentially fatal diseases such as asthma, psoriasis, and jaw osteomyelitis, especially in people with compromised immune systems (Liu et al., 1991; Gabriel et al., 2016). Despite the clear adverse effects to public health and industry associated with this fungal genus, many species of *Alternaria* are a biological resource for a variety of active compounds with potential beneficial application. One such compound that has recently been identified in *Alternaria* is resveratrol (Shi et al., 2012). However, the pathways and molecular mechanisms involved in the production of the harmful and beneficial compounds within this genus remain largely unexplored in the literature.

Transcriptome sequencing has been widely used in gene annotation, marker development, complex transcriptional regulation, and active compound metabolic pathway research in the fields of medical science (Quan et al., 2015), phytology (Wang et al., 2015), and fishery science among others (Chinchilla et al., 2015). In microbiology especially, transcriptome sequencing is a powerful tool to investigate microbial interactions in mixed populations, pathogenicity, evolutionary history, pathogenesis upon host infection and the key enzymes and associated metabolic pathways of active compounds (Krajaejun et al., 2014; Irla et al., 2015). As a tool with high throughput, low cost and great output, the Illumina HiSeq 2500 sequencing platform was selected for this study. Transcriptome analysis on *Alternaria* sp. MG1 provided useful and important information for subsequent study of the secondary metabolites in this strain and their associated biosynthetic pathways. In addition, the sample of *Alternaria* sp. MG1 was produced under the optimum conditions for resveratrol production in order to construct the RNA-seq library. Therefore, it was speculated that the transcriptome sequencing database and *de novo* analysis would reveal important information regarding the different genes and their associated functions in the primary and secondary metabolite biosynthesis of this fungus, particularly resveratrol. These unigene sequences and their annotations would provide a valuable resource for investigating specific pathways, processes, and functions in *Alternaria* sp. MG1, and will allow the identification of novel genes involved in the primary and secondary metabolite synthesis pathways, and thus promote the developmental regulation of this fungus.

So far, three (PAL, 4CL, and CHS/ST) of the four key enzymes have been identified in minority microorganisms respectively. There was little published regarding these key enzymes, but some genes have been recorded in the genebank. For example, PAL in *Rhodotorula glutinis* (KF770992.1) and *Trichosporon asahii* (XM014326038.1); 4CL in *Rasamsonia emersonii* (XM013469671.1) and *Colletotrichum gloeosporioides* (XM007280559.1); and CHS/ST in *Marssonina brunnea* (XM007294196.1) and *Aspergillus flavus* (XM002380742.1). However, no complete pathway for microorganisms has been predicted. In this study, a total of 115 pathways represented by a total of 2701 unigenes were predicted in *Alternaria* sp. MG1. The predicted metabolic pathways in this strain contributing to resveratrol biosynthesis were that of glycolysis/gluconeogenesis (ko00010), phenylalanine, tyrosine, and tryptophan biosynthesis (ko00400), phenylpropanoid biosynthesis (ko00940), and stilbenoid, diarylheptanoid, and gingerol biosynthesis (ko00945). Overall, 20 unigenes encoding four enzymes were annotated in the resveratrol biosynthesis pathway. This work not only presented the first *de novo* transcriptome sequencing analysis of RNA from *Alternaria* sp. MG1, but also revealed the possible candidate genes of relative pathways in this fungus for the first time. Two unigenes were successfully located in a cluster of chalcone synthase and stilbene synthase by phylogenetic analysis, with high sequence homology within the CHS-like domain of active and product binding sites. In addition, we obtained abundant transcriptomic data for *Alternaria* sp. MG1, and carried out an integrated analysis on the gene expression of resveratrol biosynthesis. These results can be used to discover the genetic foundation of *Alternaria* sp. MG1, especially the resveratrol biosynthetic pathway, as well as the synthesis pathways of other active compounds. This understanding could pave the way for genetically improved varieties of *Alternaria* sp. MG1 with increased secondary metabolite yields.

More importantly, the putative transcriptome information obtained in this study will provide a significant contribution toward understanding resveratrol metabolism and biosynthesis and may help in facilitating elucidation of secondary metabolite molecular mechanisms in other microorganisms.

## Ethical approval

This article does not contain any studies with human participants or animals performed by any of the authors.

## Author contributions

JC, the author of this paper was in charge of this study, including the experimental design and implementation, drafting the article, etc. JS, the corresponding author of this paper was supervising the Work. ZG, a member of this team. Assist to references collection, data analysis, article proofreading, etc. YZ, a member of this team. Assist to complete the experiment, data analysis, article proofreading, etc.

## Funding

This project was supported by the National Science-technology Support Plan Projects (No. 2015BAD16B02), the National Natural Science Fund (Grant No. 31471718), the Agriculture Department of China (Grant No. CARS-30), and the Northwestern Polytechnical University (Grant Nos. 3102014JCQ15011, 201410699086, and 3102014GEKY1010).

### Conflict of interest statement

The authors declare that the research was conducted in the absence of any commercial or financial relationships that could be construed as a potential conflict of interest.

## Footnotes

^1^http://trinityrnaseq.github.io/

^2^http://www.genome.jp/tools/kaas

^3^http://web.expasy.org/translate/

^4^http://www.megasoftware.net/mega.html

^5^http://www.softpedia.com/get/Science-CAD/GeneDoc.shtml
